# Challenges of Hospital Oxygen Management during the COVID-19 Pandemic in Rural Nepal

**DOI:** 10.4269/ajtmh.21-0669

**Published:** 2022-02-18

**Authors:** Navin Bhatt, Shristi Nepal, Richard J. Pinder, Don Eliseo Lucero-Prisno, Shyam Sundar Budhathoki

**Affiliations:** ^1^ B. P. Koirala Institute of Health Sciences, Dharan, Nepal;; ^2^ Sukraraj Tropical and Infectious Disease Hospital, Teku, Kathmandu, Nepal;; ^3^ Department of Primary Care and Public Health, School of Public Health, Imperial College London, London, United Kingdom;; ^4^ Department of Global Health and Development, London School of Hygiene and Tropical Medicine, London, United Kingdom;; ^5^ Nepalese Society of Community Medicine, Lalitpur, Nepal

## Abstract

Oxygen support remains essential for treatment of acute and severe manifestations of COVID-19. In Nepal, like many other low-resource settings, medical oxygen availability was inadequate before the pandemic. The mid-2021 wave of COVID-19 transmission starkly exposed the supply–demand imbalance of medical oxygen across the country. Pre-pandemic, more complex cases were typically referred to hospitals with better resources; however, during the pandemic, these hospitals were overrun. Therefore, resource-poor health facilities have been attempting to provide greater levels of care. However, we are faced with numerous challenges to provide a proper oxygen supply in these health settings. At a logistical level, complex geographies, sparse infrastructure, and inadequate electricity supply pose challenges. On a provider level, a shortage of trained staff and equipment necessary to administer and monitor medical oxygen creates additional pressures. Recognizing the end of the pandemic is still a long way off in many parts of the world, it is imperative that scalable, sustainable approaches to provisioning oxygen to those in greatest need are considered at a policy level.

## INTRODUCTION

Oxygen supplementation is an integral part of the management of various respiratory diseases. Its importance has been highlighted during the COVID-19 pandemic. Both acute and severe manifestations of COVID-19 are managed with oxygen. ^1^ Supply of oxygen can take various forms: oxygen cylinders, oxygen concentrators, or liquid oxygen. ^1^ Despite most people experiencing mild or uncomplicated symptoms, approximately 15% of patients diagnosed with COVID-19 require oxygen support. ^2^

During the mid-2021 wave of the pandemic, for the estimated half a million people infected with COVID-19 in low- and middle-income countries (LMICs), 1.1 million cylinders per day were needed. ^3^ Despite this, a large number of patients did not have access to oxygen. ^3^ Shortage of medical oxygen has been reported by LMICs, primarily from South America, Africa, and Asia, where oxygen demand has increased 100- to 200-fold during this pandemic. ^4^ COVID-19-related mortality rates among LMICs are as high as 19% in Yemen, ^5^ which also reported a medical oxygen shortage crisis. ^6^^,^ ^7^

In low-resource settings, including Nepal, the surge of COVID-19 has starkly exposed the gap between oxygen supply and demand. Oxygen is available in limited quantities in Nepal, and the unequal distribution of available oxygen between urban and rural areas has led to increased unmet needs for oxygen by patients in rural areas. ^8^ We aim to draw attention to the challenges faced by rural medical institutions and communities in Nepal during the COVID-19 pandemic, describe the gap between oxygen supply and demand in Nepal’s rural areas, and generate discussions regarding durable solutions to oxygen supply problems for current and future crises in low-income settings.

## HEALTH SYSTEM IN RURAL AREAS OF NEPAL

Access to basic health-care services in geographically remote and complex areas of Nepal is challenging. Health posts, which account for 78.3% of total public health-care facilities, are operated by paramedical personnel without doctors. ^9^ Patients with complex conditions are directed to primary health-care centers, which have doctors but minimal other resources. As a result, patients who require more advanced care are routinely sent to tertiary care facilities. ^10^ In Nepal, there are just 125 public health institutions with tertiary care services; most of them are located in urban areas. ^9^ The WHO’s recommended doctor-to-population ratio is 1:1,000. ^11^ However, in rural Nepal, this ratio is one doctor for every 150,000 people. ^12^ Furthermore, the extensive distance and the associated time needed in rural areas to access health facilities discourages service use, ^13^ driving a range of health inequalities. As a result, it is clear that individuals in rural regions are severely deprived of quality health-care services in terms of skilled staffing and infrastructure.

## OXYGEN SUPPLY NEEDS IN RURAL AREAS

For its population of 29 million, the country has only 1,127 intensive care unit beds and 1,555 high-dependency unit beds. ^14^ Across the entire country, only 453 ventilators are available. ^14^ These infrastructures are mostly concentrated in metropolitan areas. Hence, referrals from rural areas for oxygen support and complicated cases are very frequent. Despite equipment such as oxygen concentrators and ventilators being made available in some remote places by Nepalese local and central governments, the take-up has been limited in such areas because of the lack of trained staff and resources, including lack of technical support to sustain use and maintenance. ^15^ The lack of a reliable, continuous electrical power supply is also a challenge for mechanical life support systems. Attempts to use local generators are constrained by the availability of timely fuel supplies. ^16^

Most commercial oxygen plants are located in central Nepal and major cities. Currently, only 14% of the hospitals in Nepal have oxygen plants, and of those that have them, not all plants are operational. ^17^ Hospitals with oxygen plants also suffer from a major waste of oxygen as a result of leakages. ^8^ In the most deprived rural areas, transporting the required oxygen is another major issue. In such scenarios, people are often bound to carry oxygen cylinders by hand, and walk long distances from cities to their local health-care facilities on foot. Road ambulances and helicopters (if available) for transporting oxygen are prohibitively costly and often necessitate families to take out loans to cover costs.

## COVID-19 AND ITS IMPACT ON HEALTH CARE IN RURAL NEPAL

The surge of COVID-19 cases has exposed longstanding vulnerabilities of the Nepalese health system. As the number of COVID-19 cases surged, the health-care facilities were swamped with patients. ^18^ To make things worse, even the specialist hospitals had shortfalls in oxygen supply for the in-patients. In Kathmandu, the capital of Nepal, only 9,000 oxygen cylinders a day were available in the context of an estimated demand of approximately 30,000 cylinders. ^8^ As a result, patients’ families were forced to set out in search of sourcing medical oxygen, ^19^ and waivers of liability were introduced by health-care providers unwilling to be held liable if patients deteriorated from insufficient oxygen provision. The news of 13 in-patients requiring oxygen supplementation who died in the same day as a result of the exhaustion of the oxygen supply escalated public panic. ^20^^,^ ^21^ Consequently, the market responded with overpricing, mass stocking, and black-marketing of oxygen cylinders that restricted the availability of oxygen cylinders even to those in desperate need. ^22^^,^ ^23^

Not surprisingly, the sparse local health-care system in rural Nepal was rapidly compromised as precarious physical infrastructures and insufficient staffing were stretched beyond their limits. Because tertiary hospitals were overburdened, a referral from these rural facilities was no longer an option. ^10^ Even the complicated cases were deprived of oxygen support. The oxygen supply problems that existed before the pandemic were magnified by the pandemic.

## WHAT IS BEING DONE?

Amid the latest peak of this pandemic, it is even more unseemly that the country is experiencing political instability. ^24^ Nevertheless, there have been some commendable efforts to mitigate the impact the pandemic has caused. The donation of oxygen cylinders, concentrators, and ventilators from various humanitarian organizations and international communities has been a rare source of solace in an otherwise deeply distressing situation. In addition, the government of Nepal has issued a tax exemption on oxygen cylinders. ^25^ Nonprofit organizations such as Nepal’s National Innovation Center are supporting hospitals and health-care workers to restore malfunctioning or out-of-condition oxygen equipment, and designing oxygen delivery systems and distributing them free of charge. ^26^ In addition, climbers have been asked to bring back their empty oxygen tanks from Everest so they can be refilled and used in hospitals. ^4^ However, these efforts were targeted to mitigate the acute shortage in oxygen supply mostly in urban areas, whereas rural areas were still under-prioritized.

## WHAT MORE CAN BE DONE?

As a sustainable approach for a crisis like this in LMIC settings, efforts should focus on looking at the issues from the health systems approach. Looking through the WHO health system building blocks framework, the oxygen demand–supply crisis needs attention in four of the six areas as described next. ^27^

The first area includes medical products. One of the ways to ensure oxygen plants in hospitals generate oxygen at the point of use is by pressure swing adsorption oxygen plants. ^28^ These can be transported and installed onsite in hospitals in the form of systems, which are quite compact. Although requiring a large, upfront operational investment, pressure swing adsorption plants are a sustainable local solution that can be achieved by developing partnerships among local governments and the private sector.

Health information is a second area that needs attention. It is imperative that we strengthen the existing health information system to monitor oxygen requirements and availability effectively to ensure timely external response when oxygen demands increase.

The third area is health-care financing. Government budgeting systems need to focus on effective, sustainable investments in systems that can enable oxygen production at local levels in hospitals, and at manufacturing plants at provincial levels, that can supply oxygen when the local system is under pressure and in need.

Leadership/governance is the fourth area that needs attention. The overarching building block is leadership to ensure adequate political commitment at the national and local levels for policy formulation/implementation, including budgeting and logistics management of oxygen as an essential medical supply. Mobilization of resources at local levels ^29^ are also required, as is facilitation of the formulation of policies.

Collectively, these efforts help ensure the facilitation of economic, sustainable supply chains for remote health-care facilities by standardizing the rates for ambulances and helicopter airlifts. The sourcing and development of transportable and sustainable oxygen equipment such as mobile oxygen concentrators should be prioritized along with an alternate means of providing an uninterruptible supply of electricity, such as solar-powered oxygen delivery systems. ^30^ Focusing on the mobility and agility of oxygen equipment is another area for investigation—considering battery-powered oxygen concentrators, lightweight oxygen cylinders, and efficient delivery devices such as double-trunk masks. ^1^

## CONCLUSION

The pandemic has cruelly exposed the vulnerabilities of Nepal’s health system, of which the supply of oxygen is but one very important and concrete example. The challenge of providing health care amid resource constraints and complex geography demonstrates the centrality of supply chain logistics to safe and effective care. A thorough evaluation of existing supply management systems using frameworks such as the WHO health system building blocks may help to develop effective and sustainable oxygen delivery chains, encompassing both physical material and provider-side technical capability. We are facing both a challenge and an opportunity to learn important lessons from the COVID-19 pandemic and its impact on hospital oxygen management in Nepal.
